# Size is not everything: rates of genome size evolution, not *C*-value, correlate with speciation in angiosperms

**DOI:** 10.1098/rspb.2015.2289

**Published:** 2015-12-07

**Authors:** Mark N. Puttick, James Clark, Philip C. J. Donoghue

**Affiliations:** School of Biological Sciences, University of Bristol, Bristol Life Sciences Building, 24 Tyndall Avenue, Bristol BS8 1TQ, UK

**Keywords:** angiosperms, genome size, evolvability, polyploidy, genome duplication

## Abstract

Angiosperms represent one of the key examples of evolutionary success, and their diversity dwarfs other land plants; this success has been linked, in part, to genome size and phenomena such as whole genome duplication events. However, while angiosperms exhibit a remarkable breadth of genome size, evidence linking overall genome size to diversity is equivocal, at best. Here, we show that the rates of speciation and genome size evolution are tightly correlated across land plants, and angiosperms show the highest rates for both, whereas very slow rates are seen in their comparatively species-poor sister group, the gymnosperms. No evidence is found linking overall genome size and rates of speciation. Within angiosperms, both the monocots and eudicots show the highest rates of speciation and genome size evolution, and these data suggest a potential explanation for the megadiversity of angiosperms. It is difficult to associate high rates of diversification with different types of polyploidy, but it is likely that high rates of evolution correlate with a smaller genome size after genome duplications. The diversity of angiosperms may, in part, be due to an ability to increase evolvability by benefiting from whole genome duplications, transposable elements and general genome plasticity.

## 1. Introduction

Evolutionary biology has long sought to explain the uneven diversity across the branches of the tree of life. The land plants (Embryophyta) are a focal example, with approximately 320 000 species known, 268 600 are angiosperms [1]; indeed, the immediate sister lineage of angiosperms can muster only approximately 1050 species [1]. Many factors have been used to explain this imbalance, such as environmental opportunity [2] and key adaptations [3,4], whereas recent attention has been focused on genome size [5–7].

Across the tree of life, genome size has been linked causally to increased diversification. Traditionally, larger genomes have been linked to greater rates of speciation, but there is also evidence of smaller genomes promoting diversification, including in plants [8–10]. Furthermore, many factors relating to genome size are related to higher diversification in plants: whole genome duplication [5,11–18], transposable elements [7] and selective pressures can cause differences in genome size and diversification [10]. Theory and some experimental evidence suggests a role for genome size in variations of diversification rates, but much attention has so far has concentrated upon the size of genomes, yielding equivocal results [10].

Angiosperms are exceptional in their approximately 2000-fold variation in genome size, which has been linked to their successful diversification [5,19,20]. This contrasts strongly with the narrow variance in the larger genomes of gymnosperms [5,12,21,22]. Many factors related to evolvability are expected to alter genome size, but not unidirectionally towards a larger or smaller size [23]. Therefore, rates of size change, not absolute size, of genomes, are likely to be an important factor in explaining the differing rates of diversification across land plants.

High rates of trait evolution are associated with increased diversification potential across the tree of life [24,25]. High rates of genome size evolution promoting higher diversification in angiosperms are compatible with this hypothesis. Two main theories could explain a positive relationship between the two: punctuated evolution, in which the majority of phenotypic change occurs at speciation [26,27], especially in plants where there is a high incidence of polyploidy [28], or some form of ‘evolvability’, in which the capacity to change phenotype allows for higher rates of speciation [24,25]. However, differentiating punctuational models from evolvability models can be difficult [29], and it is likely the two are not mutually exclusive.

Genome size evolution can be modelled as a trait on a phylogenetic tree, and this allows for testing of the correlation between the rates of diversification and genome size evolution [30,31]. Here, we test this relationship across land plants using a large database of genome sizes, and predict a positive correlation between high rates of genome size evolution and speciation across the phylogeny, particularly in the angiosperms, but expect no relationship with genome size and speciation. We find this relationship to be true, with particularly high levels of size evolution in the eudicots and monocots, particularly the grasses (Poaceae). The ability to rapidly change genome size may have increased the evolvability of angiosperms, and allowed them to diversify spectacularly.

## 2. Methods

The most comprehensive, dated phylogeny of land plants [32] was used to model genome size evolution. When genome size data were considered, the phylogeny was pruned down to 3351 species of land plants.

We obtained genome sizes (1C, picograms) from the Kew *C-*value database [19]. Although we term 1C as ‘genome size’ here, we recognize the true definition is of 2C divided by the level of ploidy [30,33].

### (a) Rates of speciation and genome size evolution

Bayesian analysis of macroevolutionary mixtures (BAMMs) was used to analyse genome size evolution and rates of speciation separately on the phylogeny [25,34]. BAMM allows for multiple rate shift configurations to be modelled on phylogenies, thus it is not dependent upon a single shift configuration. Rate shifts are modelled via a compound Poisson process [34], and so no priors are required on the location of rate shifts. Diversification is modelled using parameters to represent speciation and extinction, and trait evolution is modelled as a Brownian motion process [25,34].

Priors for the reversible-jump mcmc model in BAMM were estimated using BAMMtools [35] in the software package R [36]. BAMM was run for 400 million generations for the phenotypic data, and 40 million for the analyses of speciation. Convergence was judged upon parameters exceeding 200 estimated sample size; this was more than 1000 for most parameters in the phenotypic data and analyses of speciation.

To incorporate non-random incomplete sampling, we followed established BAMM protocols. We assigned each species to a monophyletic family and calculated the proportion of species present in each family, as well as the overall proportion of land plant species. We obtained information about the number of valid species, as well as total plant species, from the plant list [37].

### (b) Correlation between rates of genome size evolution and speciation

Correlation between the rates of genome size evolution and speciation within 276 embryophyte families [25], and rates were estimated for higher-level clades. The second was to study correlations between the rate of phenotypic evolution and family diversity, in terms of species richness [38,39]. We also tested whether size was correlated with speciation rates across the tree using traitDependent BAMM, which is a method that computes correlation coefficients between the trait and random posterior speciation rates from BAMM samples.

Phylogenetic generalized least-squares (GLS) models were used to account for the effects of phylogeny in the regression of speciation rates on rates of genome size evolution [25,39–41]. PGLS models were based on code from the CAPER package in R [42]. PGLS quantifies and incorporates similarity between species owing to the shared phylogenetic history by estimating Pagel's *λ* [40,43]—this similarity is then incorporated into the error term of the regression model [44].

As we tested the correlations of two rates, both could be positively correlated with time [25]. Therefore, we also tested for evidence of this relationship by looking at the influence of time by examining the rates between sister-clades only which, by definition, are of equal age [25].

### (c) Direction of change

We used StableTraits [45] to estimate ancestral sizes of genomes throughout the phylogeny. StableTraits samples rates from a heavy-tailed [45,46], rather than a normal distribution, as in Brownian motion [47]. This allows for rate changes to be estimated parametrically on the tree, such that individual branch rates and ancestral node estimates can be calculated for the entire tree. StableTraits was run for 80 million generations, sampling at every 1000 generations, and across two independent chains.

## 3. Results

### (a) Rates of speciation and genome size evolution

Speciation and genome size evolution show considerable variation throughout the phylogeny. In the model of genome rate evolution, the mean log-likelihood of the posterior was 3583.77 (3426.84–3740.07, 2.5 and 97.5 percentiles, respectively) and the mean number of shifts was 62 (56–69, 2.5 and 97.5 percentiles, respectively). Similar results were found for rates of speciation: the mean number of shifts was 48 (39–58, 0.025 and 0.975 quantiles, respectively), and the mean log-likelihood of the posterior was −11 534.65 (−11 674.6 to −11 448.8, 02.5 and 97.5 percentiles, respectively). Although it was not possible to calculate Bayes factors—the prior was zero for many of the shifts—there is a clear difference between the prior and posterior for the number of shifts (see electronic supplementary material, figures S1 and S2).

**Figure 1.**
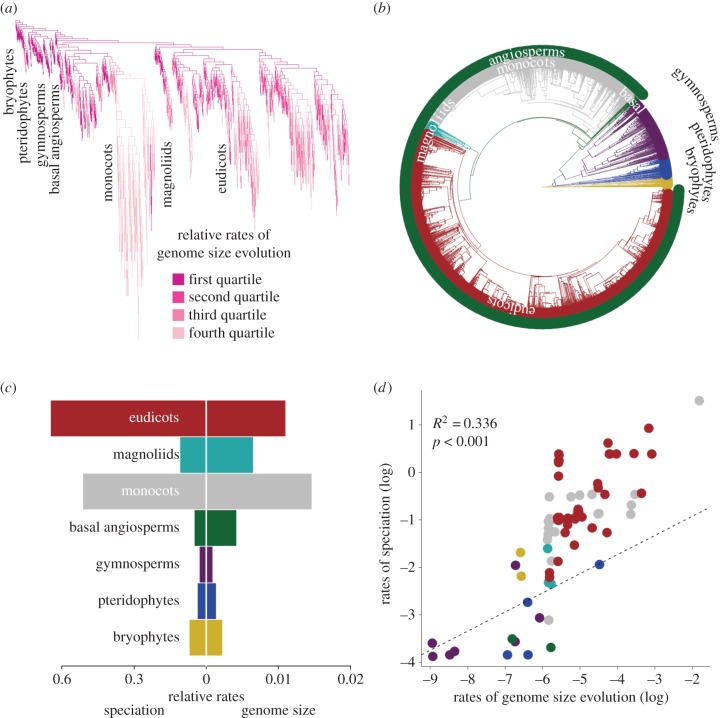
Rates of speciation and genome evolution are correlated in plants. The highest rates of speciation (branches scaled to rate) are associated with the highest genome rates (coloured branches) (*a*). Clades shown in the phylogeny (*b*) show correlation between rates of genome size evolution and speciation (*c*), and there is a significant relationship in a phylogenetically corrected correlation between the two rates for families (*d*).

Angiosperms show the highest rates of genome size evolution and speciation (table 1 and figure 1). Mean clade rates in the angiosperms for speciation (0.55) and genome size evolution (0.009) were higher compared with the speciation rate (0.04) and genome size evolution rates (0.001) in non-angiosperms. Within angiosperms, very high rates of genome size evolution are found within monocots (figure 1), particularly Poaceae (0.16), which also exhibits the highest rate of speciation (4.53). The lowest rates of speciation (0.03) and genome evolution (0.03) are found in gymnosperms. The families Pinaceae (0.0001) and Araucariaceae (0.02) have the lowest speciation and genome rates, respectively (see electronic supplementary material, figure S4).

**Table 1.** Rates of speciation and genome size evolution for clades in the phylogeny.

	speciation rate	genome rate
angiosperms	0.55 (0.48, 0.62)	0.009 (0.008, 0.01)
non-angiosperms	0.04 (0.03, 0.07)	0.001 (0.001, 0.001)
bryophytes	0.07 (0.04, 0.18)	0.002 (0.0009, 0.004)
pteridophytes	0.04 (0.03, 0.06)	0.001 (0.0006, 0.002)
gymnosperms	0.03 (0.02, 0.04)	0.0007 (0.0003, 0.002)
basal angiosperms	0.05 (0.02, 0.27)	0.003 (0.002, 0.005)
magnoliids	0.11 (0.05, 0.33)	0.005 (0.002, 0.01)
monocots	0.51 (0.42, 0.65)	0.011 (0.009, 0.01)
eudicots	0.65 (0.52, 0.72)	0.008 (0.007, 0.009)

### (b) Positive correlation between rates of genome size evolution and speciation

At the family level, there is a significant relationship between rates of genome size evolution and speciation across the tree (figure 1). The PGLS model, which tests for the significance of the relationship at the family level (figure 1*b,d*), indicates a strong relationship between genome size evolution and speciation rates (*p* < 0.001, 90 d.f., *R*^2^ = 0.383). This is also significant within just angiosperms (*p* < 0.001, 76 d.f., *R*^2^ = 0.359) (table 2). These results are also significant when using contrasts.

**Table 2.** PGLS analyses show the positive relationship between genome size rates of evolution and speciation rates and family diversity for all plants and angiosperms only.

	d.f.	*p*-value	*R*^2^	lambda (95% CIs)
all plants				
speciation rates	90	3.02 × 10^−11^	0.3826	0.593 (n.a., 0.895)
family diversity	90	2.08 × 10^−10^	0.3565	0 (0, 0.408)
angiosperms only				
speciation rates	76	1.62 × 10^−8^	0.336	1 (0.874, n.a.)
family diversity	76	9.19 × 10^−6^	0.2192	0 (0, 0.496)

As an analogous test, the relationship between tip diversity of families (*n* species) and rates of genome size evolution was performed. This was very significant for the entire tree (*p* < 0.001, 90 d.f., *R*^2^ = 0.357) and within just angiosperms (*p* < 0.001, 76 d.f., *R*^2^ = 0.219; table 2 and electronic supplementary material, figure S3*a*,*b*).

Independent contrast also gave similar results to PGLS with a significant relationship between the genome size and speciation rates (*p* < 0.001, rho = 0.61). Time does not appear to be a confounding factor as contrasts between sister-species only was non-significant using the Spearman rank test (*p* = 0.054). While this is used to test the confounding effect of time on analyses [25], it is likely that our negative result here is due to the small sample size (*n* = 28), and there is still a positive relationship (rho = 0.37). Furthermore, gymnosperms and angiosperms are the same age, by definition, and show no evidence of correlation in rates.

**Figure 2.**
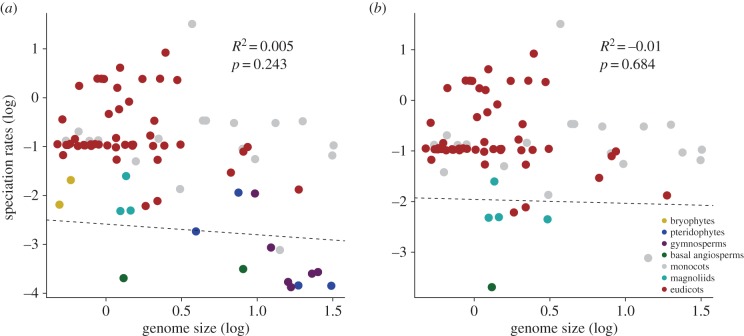
There is no significant relationship between overall genome size and rates of speciation for all land plants (*a*), and just angiosperms (*b*) when using a PGLS regression at the family level. Permutation tests also show a non-significant relationship between genome size and speciation for all plants.

There is no evidence for high rates of speciation being linked to genome size (as opposed to *rates* of genome size evolution; figure 2). We find no significant correlation between overall speciation rates and genome size for the entire tree (*p* = 0.243, 83 d.f., *R*^2^ = 0.005), or angiosperms (*p* = 0.68, 76 d.f., *R*^2^ = −0.01). traitDependentBAMM also shows a non-significant correlation between genome size and speciation rates across the tree (*p* = 0.56).

**Figure 3.**
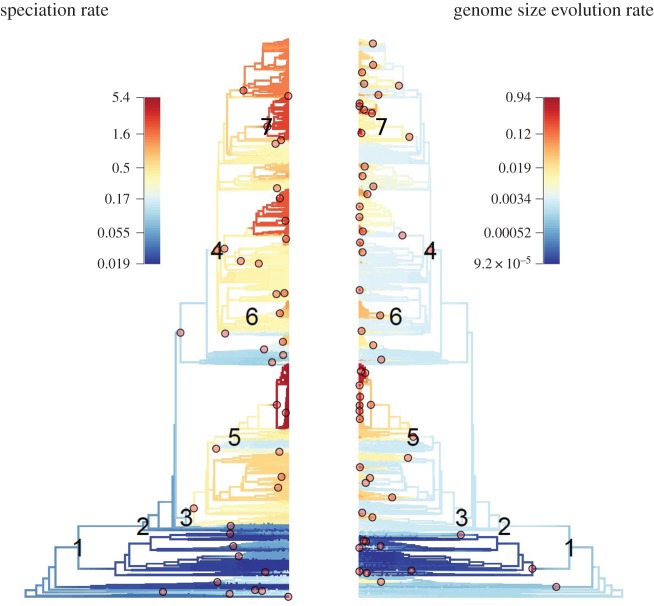
The position of shifts for rates of speciation and genome size evolution on the phylogeny compared with reported whole genome duplications in the Spermatophyta (1), Angiospermae (2), monocots (3), eudicots (4), Poaceae (5), Brassicaceae (6) and the Asteraceae (7). Only the core eudicots (4) show accelerated rates for speciation and genome size evolution.

We find little evidence for accelerations on branches leading to the major clades of angiosperms at sites associated with whole genome duplications. Rates on branches leading to angiosperms (0.003), monocots (0.002) and eudicots (0.003) all fall into the first quartile of rates throughout the phylogeny. Furthermore, there is little evidence to link purported whole genome size changes and accelerated rates of speciation or genome size evolution. We plotted the posited location of whole genome duplication events on the phylogenies displaying the best shift configurations of diversification and genome size evolution, respectively (minimum Bayes factor 5); these results indicate that only the core eudicots are associated with a shift in speciation and trait evolution rates (figure 3). Other whole genome duplication events are not associated with differences in speciation and trait evolution rates of evolution.

### (c) Ancestral states and the direction of change

**Figure 4.**
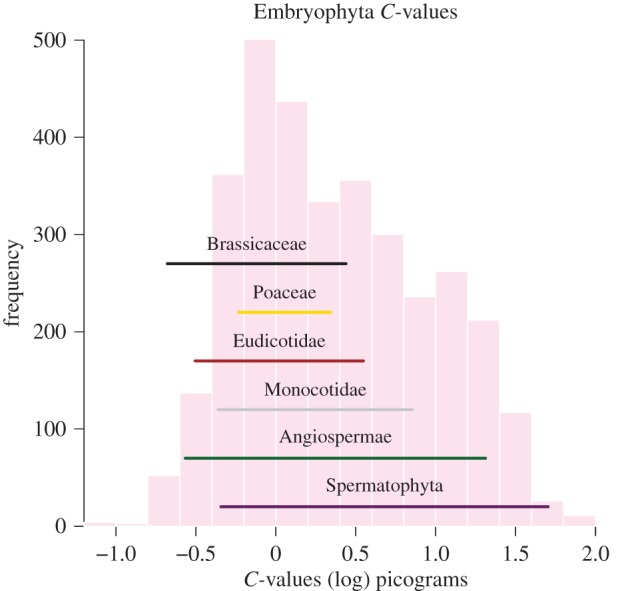
There is large uncertainty in ancestral reconstruction of genome size for nodes associated with whole genome duplication events. The histogram shows known *C*-values from extant land plants, and the coloured lines represent the range of uncertainty (95th% highest posterior density) for ancestral reconstruction of genome size in the common ancestors of crown clades.

The reconstructed ancestral angiosperm genome size is 1.45 picograms (0.57–3.71 95% highest posterior density) which is smaller than the size estimated for the ancestral spermatophyte of 1.99 picograms (0.7105.49 95% highest posterior density; see electronic supplementary material, table S1 and figure 4). As expected, high rates of genome size evolution are associated with increases and decreases in *C*-value throughout the tree; there is no difference in the distribution of size changes in ancestor–descendant pairs between angiosperms and non-angiosperms (*p* = 0.1531, Wilcoxon rank-sum test). Therefore, it appears increased rates are associated with both increases and decreases in *C*-value throughout the phylogeny.

## 4. Discussion

While genome size has been traditionally linked to the success of angiosperms, here we find that it is the ability to alter genome size that exhibits the strongest correlation with diversity. This fits a hypothesis in which genome size in and of itself is not an important factor for diversification as has been previously suggested [10], but it is the ability to cope with genome size changes that has allowed angiosperms to benefit from polyploidy and other genome rearrangements [5,8,9,12,48]. Changes in genome size are likely to have promoted diversification in angiosperms, especially compared with the species-poor gymnosperms [22].

As expected, the large variance in *C*-value for angiosperms [5,12,49] translates into a high rate of genome size evolution, and this correlates strongly with rates of speciation (figure 1). A frequent explanation for the huge diversity of angiosperms is the prevalence of whole genome duplication events [5,20]. However, directly linking *C*-value to polyploidy events can be difficult: *C*-value is not directly proportional to ploidy and often downsizes following duplication [50,51]. As we measure changes in *C*-value, these are very likely to be influenced by whole genome duplications as well as other factors linked to increased rates of diversification, such as tandem duplications, transposable elements ([7,47], but see [52]), life history [53] and deletions [8,51,54]). As a guide to ‘genome size’, *C*-value effectively captures large-scale patterns in genome size change throughout the phylogeny, but it is not attributable to one effect, such as whole genome duplications, alone. Overall, we support a model in which higher rates of genome size evolution that result from range of processes promote higher rates of speciation [7] (figure 1).

### (a) Evolvability

High rates of genome size evolution correlate with high rates of speciation in angiosperms, and confirm previous predictions that genome size variability is linked to success in flowering plants [5]. These patterns could fit a punctuational model of evolution in which genome size changes occur at speciation [26], or a model of evolvability in which higher rates of genome change drives high rates of speciation [7,24,25]. Discriminating among punctuational and evolvability models is not trivial [29], and we cannot reject the possibility that they are linked, but this does not require one model being favoured at the expense of another. A large amount of change may be expected at speciation in a punctuational model [7,26–28,55]. A subset of this model posits that genome size changes, and by definition, speciation, are associated with cladogenesis—speciation results from polyploidy, but polyploidy does not promote diversification [12,28,49]. These models would imply small genome size is a consequence of, not a driving factor behind, diversification. However, we find no link between genome size and rates of speciation (figure 2), and we expect to find a small genome size in many species that have undergone recent, rapid radiations [5,56]. Therefore, there are many reasons to associate genome size change with higher rates of speciation in an evolvability model (figure 1): whole genome duplications [13,14], via general genome plasticity [5,12,48], lowering extinction risk by reducing genome size [8], the action of transposable elements [7] and retaining benefits of duplicated genes [48]. Thus, we cannot definitively differentiate between punctuational and evolvability models, but we suggest there is evidence to infer an evolvability model relating to higher rates of genome size evolution in plants (figure 1).

### (b) Whole genome duplications

In the past, authors have argued that polyploidy and duplicated elements within genomes could lead to ‘genetic obesity’ [57], but despite multiple rounds of duplication we find no evidence for directional evolution in genome size. While it has become clear that increases and decreases in genome size are characteristic of angiosperms [5,30,51], we find no relationship between absolute genome size and rates of speciation in angiosperms or in embryophytes more generally (figure 2). Out of a number of proposed genome duplications [16,58–62], only core eudicots show a consistent shifts in rate for genome size evolution and diversification (as judged by Bayes factors; figure 3), and some clades associated with ancestral polyploidy show heightened rates of diversification (monocots, eudicots, Brassiceae, Asteraceae and Poaceae). Spermatophyta and Angiospermae do not show heightened speciation or genome size evolution rates. It can be seen that not all angiosperms have experienced a heightened rate of evolution (figure 1). This might evidence a model in which early-diverging lineages, including *Amborella*, did not undergo recent rounds of whole genome duplication and so do not exhibit higher rates of speciation [63], and demonstrates how nested diversifications may follow from whole genome duplications [20]. A relatively small ancestral angiosperm genome size has been suggested [64], but here the posterior density around our estimates for ancestral angiosperms is very large (figure 4). At present, it is possible to elucidate large-scale patterns in genome size evolution, but obtaining precise ancestral estimates for angiosperms may be difficult [65,66], but promise may come through working with fossils ([67], but see also [68]).

### (c) Auto- and allopolyploidy

In this study, we do not differentiate between auto- and allopolyploidy, and the related subject of dosage-dependent and dosage-independent genes. Autopolyploidy is initially thought to maintain dosage balance via the retention of dosage-dependent genes, though over time it is thought that these may diverge in function or expression [23,69]. However, genomic rearrangements and heterosis effects are thought to be stronger in allopolyploids [69], and so it is likely to have had a large role in plant evolution, but current methods only tentatively identify a small number of differentiable auto- and allopolyploidy events (*n* = 9), and some of these are not phylogenetically positioned [69]. Thus, making statistical analysis of these events unfeasible at present, but incorporation of auto- and allopolyploidy events will improve future investigations.

## 5. Conclusion

Rates of genome size evolution are positively correlated with diversification rates in plants, a trend that is driven by largely by the positive relationship in angiosperms. No evidence supports a link between overall size and diversification. Overall, these results support a model in which rate of genome size evolution promotes the acquisition of novel traits, reproductive barriers and movement into new niches, which have aided the diversification of angiosperms.
